# The relationship between *Porphyromonas gingivalis* and oesophageal squamous cell carcinoma: a literature review

**DOI:** 10.1017/S0950268823000298

**Published:** 2023-04-03

**Authors:** Jinyu Kong, Yiwen Liu, Mengfan Qian, Ling Xing, Shegan Gao

**Affiliations:** 1 School of Information Engineering, Henan University of Science and Technology, Luoyang, China; 2 Henan Key Laboratory of Microbiome and Esophageal Cancer Prevention and Treatment, Henan Key Laboratory of Cancer Epigenetics, Cancer Hospital, The First Affiliated Hospital and College of Clinical Medicine of Henan University of Science and Technology, Luoyang, China

**Keywords:** Oesophageal cancer, oesophageal microbe, oral microorganism, *Porphyromonas gingivalis*, prognosis

## Abstract

Oesophageal cancer is the most common gastrointestinal malignancy in China and one of the major causes of death due to cancer worldwide. The occurrence of oesophageal cancer is a multifactor, multistage, and multistep process influenced by heredity, the environment, and microorganisms. Specifically, bacterial infection may be involved in the process of tissue carcinogenesis by directly or indirectly influencing tumour occurrence and development. *Porphyromonas gingivalis* is an important pathogen causing periodontitis, and periodontitis can promote the occurrence of various tumours. An increasing number of studies to date have shown that *P. gingivalis* plays an important role in the occurrence and development of oesophageal cancer. Overall, exploring how *P. gingivalis* promotes oesophageal cancer occurrence and development and how it affects the prognosis of these patients is of great importance for the diagnosis, prevention, and treatment of this type of cancer. Herein, the latest progress is reviewed.

## Introduction

Oesophageal cancer is a malignant tumour, and its prevalence is high in China. According to the statistics reported by the World Health Organization (WHO), 604,000 new oesophageal cancer patients and 544,000 deaths were reported worldwide in 2020 [1]. In China, the incidence and death rates of oesophageal cancer are 324,000 and 301,000, respectively, accounting for approximately 55% of the global total [1, 2]. The geographical distribution of oesophageal cancer cases varies greatly in China. The southern Taihang Mountain area at the border of Henan, Hebei, and Shanxi, especially Linzhou in Henan Province, represents an area with high incidence [3]. Oesophageal cancer is a serious threat to human life and health, with a 5-year survival rate of 15–25% [4]. There are two main tissue types, oesophageal squamous cell carcinoma (ESCC) and oesophageal adenocarcinoma (EAC), and ESCC is more common in developing countries [5].

Various interactive factors including sex, age, smoking, alcohol consumption, genetic factors, living environment, dietary habits, obesity, chronic viral infection, and inflammation affect the occurrence of oesophageal cancer [6]. In the study of the factors that influence malignant tumours, the subtle and unique relationship between bacteria and tumours has been neglected for many years. However, since the relationship between *Helicobacter pylori* infection and gastric cancer was confirmed in the 1990s [7], pathogenic microbial infection and microecological disorder have gradually become hot topics in tumour research. Indeed, an increasing number of studies have confirmed that specific microorganisms are associated with tumours. Allavena et al. [8] reported that approximately 20% of malignant tumours are associated with microbial infection; for example, *Epstein–Barr* virus is associated with nasopharyngeal carcinoma [9], *Fusobacterium nucleatum* infection with colon cancer [10], and *Chlamydia pneumoniae* with lung cancer [11].

Chronic periodontitis is a common chronic infectious disease of the oral cavity. An increasing number of epidemiological investigations have shown a positive correlation between periodontitis and cancer, especially oral and oesophageal cancer [12, 13]. *Porphyromonas gingivalis* is a keystone pathogen that plays a considerable role in periodontitis and is the most important pathogenic bacterium mediating the local inflammatory immune response in chronic periodontitis [14]. *P. gingivalis* exerts toxic effects of adhesion to and invasion of gingival epithelial cells, interfering with the normal physiological metabolism of cells and inhibiting programmed cell death [15]. In recent years, an increasing number of studies have shown that *P. gingivalis* is closely related to a variety of malignant tumours [16–19]. In this paper, recent research progress on the correlation and pathogenesis between *P. gingivalis* and ESCC is reviewed to provide aid for clinical practice.

The PubMed database was searched for articles relevant to the subject matter of this review, and more recently published literature was favoured. Specifically, keywords such as *Porphyromonas gingivalis*, *P. gingivalis*, oral microbiome, oral microbiota, salivary microbiota, oesophageal microbiota, oesophageal microbiome, oral health, tooth loss, tooth decay, periodontal disease, periodontitis, oral hygiene, brushing, ESCC, oesophageal cancer, digestive cancer, digestive neoplasms, orodigestive cancer, and cancer were used to generate search results. We also searched the references of selected articles. Additionally, a manual review of references from the materials found in PubMed was performed.

## Periodontitis and oesophageal cancer

It has been confirmed that chronic periodontitis can cause changes in systemic inflammation levels and is a risk factor for a variety of diseases, such as cardiovascular disease, diabetes, premature birth, and low birth weight. In recent years, periodontitis, tooth loss, and oesophageal cancer have been found to be closely related. For instance, Lo et al. [20] found in a large cohort study that individuals with a history of periodontal disease have an increased risk of EAC (hazard ratio (HR) = 1.43). In another cohort study, Nwizu et al. [13] showed that periodontal disease in postmenopausal women increases the risk of oesophageal cancer (HR = 3.28). A retrospective study by Lee et al. [21] determined that the risk of oesophageal cancer decreases after periodontal prophylaxis in men (HR = 0.54, 95% confidence interval (CI) 0.44–0.66). In 2001, Abnet et al. [22] conducted a 5.25-year prospective study using 620 ESCC samples from Linzhou city, an area with a high incidence of oesophageal cancer in China, and concluded that tooth loss is significantly positively correlated with the occurrence of ESCC. Additionally, Chen et al. [23] found that tooth loss increases the risk of ESCC (risk ratio (RR) = 1.3). A study on the Chinese population verified that poor oral hygiene is closely related to the occurrence of ESCC [24] and that poor oral habits and changes in the oral microecological environment may play an important role.

## *P. gingivalis* in the oral and oesophageal microbiome in ESCC

The human oral microbiome includes as many as 700 different species of bacteria [25], an increasing number of which can be isolated and cultured in vitro and have been named. These microorganisms constitute a complex oral microecology. Under normal physiological conditions, oral microbes maintain a dynamic balance; however, oral microecological imbalance can directly damage adjacent tissues or indirectly damage distant tissues through abnormal immune responses, leading to the occurrence and development of a variety of human diseases [26, 27]. Chronic periodontitis is an inflammatory disease caused by oral bacterial infection, and oral microorganisms play an important role in the tissue inflammation and destruction caused by periodontitis. Therefore, the correlation between periodontitis and oesophageal cancer may be associated with periodontal pathogens, and *P. gingivalis* infection and oral microecological imbalance are the main pathogenic bacteria that cause chronic periodontitis and tooth loss [28]. Accordingly, the influence of *P. gingivalis*, an important periodontitis pathogen, on oesophageal cancer has attracted the attention of researchers.

In a large case–control study, Chen et al. [29] explored the correlation between changes in oral microbiota and ESCC risk and observed differences in the relative abundance of oral bacteria in 87 ESCC patients, 63 atypical hyperplasia patients, and 85 healthy controls through 16S rRNA gene sequencing. The diversity of the oral microbiome in ESCC patients was significantly lower than that in the normal healthy controls and patients with atypical hyperplasia, and the bacteria that were significantly enriched included *Prevotella*, *Streptococcus*, and *Porphyromonas.* Similarly, Meng et al. [30] evaluated saliva samples from 30 ESCC patients in addition to 22 healthy controls. 16S rRNA sequencing results showed that *Porphyromonas*, *Streptococcus*, and *Leptotrichia* were the most enriched in ESCC saliva. Wang et al. [31] showed that *Actinomyces* and *Antopobium* were related to an increased risk of ESCC, whereas the presence of *Fusobacterium* and *Porphyromonas* was highly associated with the healthy group. In this study, since the ESCC patients had periodontitis or gingivitis, the authors selected healthy controls with periodontitis or gingivitis to control the effects of confounding factors. Therefore, the difference may not have been significant because patients with periodontitis or gingivitis themselves have a higher prevalence of ESCC than the normal population. A recent study showed that *Leptotrichia, Porphyromonas, Streptococcus, Rothia, Lactobacillus*, and *Peptostreptococcus* were more abundant in ESCC patient saliva than in healthy control saliva [32]. These findings suggest that oral microecological abnormalities can increase the risk of ESCC.

The oesophagus is close to the mouth, and the epithelium is squamous; thus, this tissue inevitably serves as a colonisation site for oral bacteria. Similar to the microbiome of the mouth, stomach, colon, vagina, and skin, the composition of the oesophageal microbiome is extremely complex [26]. Bacteria belonging to 41 genera and 95 species in 6 phyla have been identified in the normal oesophageal microbiome, and at least 100 symbiotic bacteria of different species colonise the lower oesophagus [33]. These bacteria mainly comprise gram-positive *Streptococcus*, whereas the microbiome of individuals with oesophagitis and Barrett’s oesophagus mainly comprises gram-negative bacteria [34]. Yu et al. [35] indicated that the relative abundance of oesophageal microbes correlates negatively with oesophageal squamous epithelial dysplasia. The authors found that the decreased diversity and change in the relative abundance of oesophageal microbes can promote oesophageal squamous cell atypical hyperplasia and may play an important role in the occurrence and development of ESCC. Furthermore, the oesophageal microbiota was prospectively investigated in 18 patients with ESCC and 11 patients with physiologically normal oesophagus by 16S rRNA gene profiling. The results indicated that **Aggregatibacter*, Veillonella, Parvimonas, Catonella, Streptococcus, Selenomonas, Porphyromonas,* and *non-Fusobacterium. Fus, Lautropia, Peptococcus, Fusobacterium* spp.*, Peptostreptococcus, Campylobacter, Dialister, Prevotella, Treponema,* and *Granulicatella* represent significantly enriched genera in ESCC [36]. These results suggest that bacterial infection or oesophageal microecological changes may play an important role in the progression of multistage oesophageal cancer.

Peter et al. [37] reported that microorganisms may play a role in the aetiology of oesophageal cancer. These authors conducted a prospective study involving 81 patients with ECA, 25 patients with ESCC, and a healthy control group to identify oral microbes by 16S rRNA gene sequencing. The diversity of the oral microbiota in ESCC patients was significantly reduced and the abundance of *Fustanella* correlated highly with the occurrence of EAC (95% CI: 1.01–1.46). The increased abundance of *P. gingivalis* increased the risk of ESCC, and *P. gingivalis* was associated with lymph node metastasis and short survival in ESCC patients. Kageyama et al. [38] conducted a case–control study examining the salivary microbiota in patients with different gastrointestinal tract cancers, including 12 with unspecified oesophageal cancer and their age- and sex-matched controls. *P. gingivalis* and *Corynebacterium* species were more abundant in the saliva of patients with oesophageal cancer than in healthy controls. In addition, Chen et al. [39] reported that the oral biofilms in ESCC patients contained more *Streptococcus species*, *Veillonella parvula*, and *P. gingivalis* than in healthy volunteers. Although there have been only a few studies, all of which had small sample sizes, these studies indicate that *P. gingivalis* may promote the occurrence of oesophageal cancer, and *P. gingivalis* may represent a risk factor for ESCC.

## *P. gingivalis* promotes the occurrence and development of ESCC

As the most important pathogenic bacterium in periodontal disease, *P. gingivalis* has been found to be associated with the development of oesophageal cancer in recent years. Through immunohistochemistry experiments and real-time quantitative polymerase chain reaction (PCR) detection, our team’s previous research reported in 2016 that the detection rate of *P. gingivalis* (61%) and 16S rDNA (71%) in ESCC tissues was much higher than that in adjacent tissue, whereas *P. gingivalis* and 16S rDNA were not detected in normal oesophageal mucosa tissue. In addition, *P. gingivalis* levels correlated positively with clinicopathological features, including tumour differentiation, lymph node metastasis, and TNM stage, and negatively with overall survival in ESCC patients [40]. Very similar results were obtained by Chen et al. [39], who in 2021 reported that 57% of patients enrolled in their study were infected with *P. gingivalis.* Moreover, the authors highlighted that *P. gingivalis* was associated with advanced clinical stages of ESCC and poor prognosis. In 2017, further research by our team revealed that large levels of *P. gingivalis* can be detected in ESCC tissues, but the low abundance or absence of this bacterium in cardia cancer or gastric cancer may be related to the low tolerance value of *P. gingivalis* to acidic environments. In addition, the positive rate of *P. gingivalis* detection was the highest at 48.3%, followed by the rates of *F. nucleatum* (35%) and *Streptococcus anginosus* (17.5%) detection, demonstrating that *P. gingivalis* potentially represents the predominant anaerobe in late aggressive ESCC [41]. In summary, *P. gingivalis* colonisation of the oesophagus is not only related to ESCC but also closely related to disease severity and prognosis.

In 2012, Ahn et al. [42] conducted a prospective cohort study using 7852 serum *P. gingivalis* IgG antibody-positive samples from the third National Health and Nutrition Survey of the United States from 1988 to 1994. After controlling for other variables, such as sex, age, smoking, education level, and body mass index (BMI), the researchers found that the *P. gingivalis* serum IgG antibody level correlated positively with mortality due to digestive malignancies, including ESCC, pancreatic cancer, and colorectal cancer. Thus, *P. gingivalis* is independent of periodontal disease and other factors that could cause cancer and is closely associated with digestive system malignant tumours, and the presence of *P. gingivalis* may increase mortality in cancer patients. Our team’s previous research measured and evaluated serum *P. gingivalis*-specific IgG and IgA antibody levels in 96 ESCC patients, 50 oesophagitis patients, and 80 healthy people using enzyme-linked immunosorbent assays (ELISA) in 2018 [43]. *P. gingivalis*-specific IgG and IgA antibody titres in ESCC patients were significantly higher than those in oesophagitis patients and healthy controls, and the expression levels of the two antibodies were significantly negatively correlated with the survival of oesophageal cancer patients, especially those with stage 0-II or negative lymph node metastasis [43]. Overall, *P. gingivalis* may be involved in the pathogenesis of ESCC, and the combined analysis of several *P. gingivalis* serum biomarkers is more sensitive and specific than the analysis of any single biomarker for diagnosis. Due to the continuity of the physiological structure between the oesophagus and the mouth, ESCC is more likely to be in contact with the oral flora than other parts of the digestive system. If the exact relationship between ESCC and *P. gingivalis* can be demonstrated, monitoring the development of ESCC by detecting *P. gingivalis* will have important clinical value.

## Possible mechanisms by which *P. gingivalis* promotes oesophageal cancer

Tumours may affect the abundance and diversity of microorganisms. In patients with cancer exhibiting low immunity, harmful microbes may take advantage of this condition and disrupt the normal distribution of human microbes. Conversely, alterations in microorganisms may also serve as a risk or protective factor for the tumourigenesis of various types of cancer [44]. Currently, the possible carcinogenic mechanisms of *P. gingivalis* include the production of virulence factors or active metabolites, the activation of carcinogenic signalling pathways, the promotion of abnormal immune responses, and the inhibition of apoptosis in host cells.

### *P. gingivalis* enhances the malignant abilities of ESCC cells

**Figure 1.**
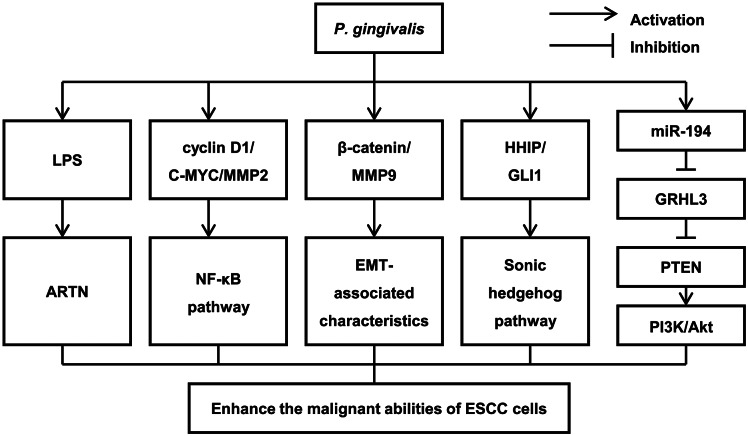
The mechanism by which *Porphyromonas gingivalis* enhances the malignant abilities of ESCC cells.

To date, studies on the mechanism by which microorganisms influence oesophageal cancer have been limited, but scholars have found that the long-term colonisation of *P. gingivalis* may be involved in the occurrence and development of ESCC. Rousseau et al. [45] reported that LPS can increase the migratory ability of human oesophageal cancer cells by increasing their adhesive properties through Toll-like receptor 4(TLR4) signalling and selectin ligands. Zhang et al. [46] identified *P. gingivalis* lipopolysaccharide (LPS) as a potential carcinogenic factor for ESCC that affects cell proliferation and migration. The mechanism may involve influencing the biological behaviour of ESCC cells by increasing the expression of ARTN. Meng et al. [30] revealed that *P. gingivalis* upregulates the expression of key molecules involved in the NF-κB signalling pathway in vitro, such as cyclin D1, matrix metalloproteinase 2 (MMP2), and C-MYC, promoting the proliferation, migration, and invasion of ESCC cells. Liang et al. [47] used high-throughput sequencing to show that miR-194 was significantly upregulated and its direct target GRHL3 was decreased after *P. gingivalis* infection. Since tumour suppressor gene PTEN is a direct regulatory target of GRHL3, GRHL3 has a positive regulatory effect on it in vivo and can regulate the occurrence and development of tumours by activating the PI3K/Akt pathway. Moreover, Chen et al. [39] found that *P. gingivalis*-infected ESCC cells exhibited enhanced EMT-associated characteristics, including elevated β-catenin and matrix metalloproteinase-9 (MMP9) expression levels, which was accompanied by reduced E-cadherin expression levels. Importantly, *P. gingivalis* infection increased the incidence of developing invasive carcinoma in a mouse oesophageal tumour model induced by 4-nitroquinoline 1-oxide. A recent article by Jia et al. [48] revealed that in the presence of *P. gingivalis*, normal human oesophageal epithelial cell proliferation and migration increased, and aneuploid cells appeared. This study indicated that *P. gingivalis* can induce the malignant transformation of normal oesophageal epithelium by activating HHIP and GLI1, which are key genes in Sonic hedgehog pathways. This study provides direct causal evidence for the carcinogenic effect of a periodontal pathogen on the normal oesophagus. The above results confirmed that *P. gingivalis* and its virulence factors could enhance the malignant abilities of ESCC cells by activating multiple signal transduction pathways (Fig. 1).

### *P. gingivalis* induces tumour cell evasion of the host immune response

**Figure 2.**
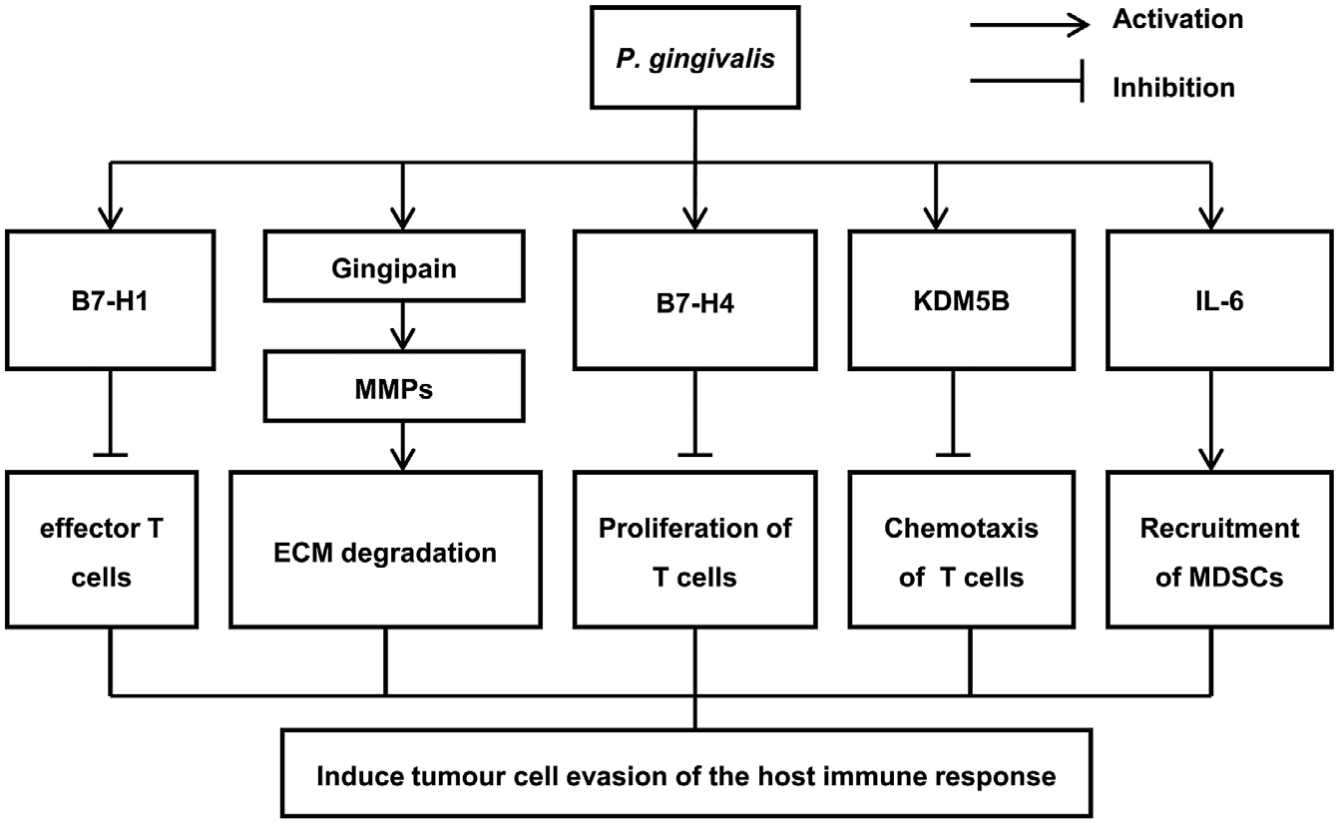
The mechanism by which *Porphyromonas gingivalis* induces tumour cell evasion of the host immune response.

To date, there have been few studies on the immune escape of tumour cells induced by *P. gingivalis*, and those that have been conducted have mainly focused on the expression of immune coregulatory receptors and immune checkpoint molecules (Fig. 2). *P. gingivalis* can induce the expression of B7-H1 and B7-DC receptors on the surface of gingival epithelial cells and squamous cell carcinoma cells. B7-H1 enhances the production of regulatory T cells, which can inhibit the function of effector T cells and promote immune avoidance in tumour cells [49]. Gingipains produced and secreted by *P. gingivalis* induces the expression of pro-inflammatory mediator, as matrix metalloproteinases (MMPs), degrades extracellular matrix (ECM), destroys immunoglobulin and complement components C3 and C5, and enables ESCC to escape killing by macrophages and neutrophils, leading to ESCC occurrence and development [50]. From the perspective of the tumour immune microenvironment, our previous study found that *P. gingivalis* colonisation in oesophageal cancer cells induces strong expression of the immune checkpoint molecule B7-H4 and lysine demethylation 5B (KDM5B), thereby promoting the evasion or inhibition of the host immune response by tumour cells, with sustained colonisation [51]. The dual factors of infection and tumours aggravate the suppression of host immunity and the weakening of immunogenicity. The study further explored the novel mechanisms of immune escape by tumour cells promoted by *P. gingivalis*, providing a new target for immune checkpoint inhibitors in the treatment of oesophageal cancer and a new strategy for immunotherapy of oesophageal cancer based on microecology. Chen et al. [39] reported that *P. gingivalis* infection induces interleukin (IL)-6 expression in ESCC cells and significantly elevates IL-6 levels in cell culture supernatants. In addition, *P. gingivalis* infection regulates the local ESCC microenvironment by promoting the recruitment of myeloid-derived suppressor cells (MDSCs), thus enabling cancer cells to escape the host immune response.

### *P. gingivalis* inhibits apoptosis in host cells and accelerates the cell cycle

**Figure 3.**
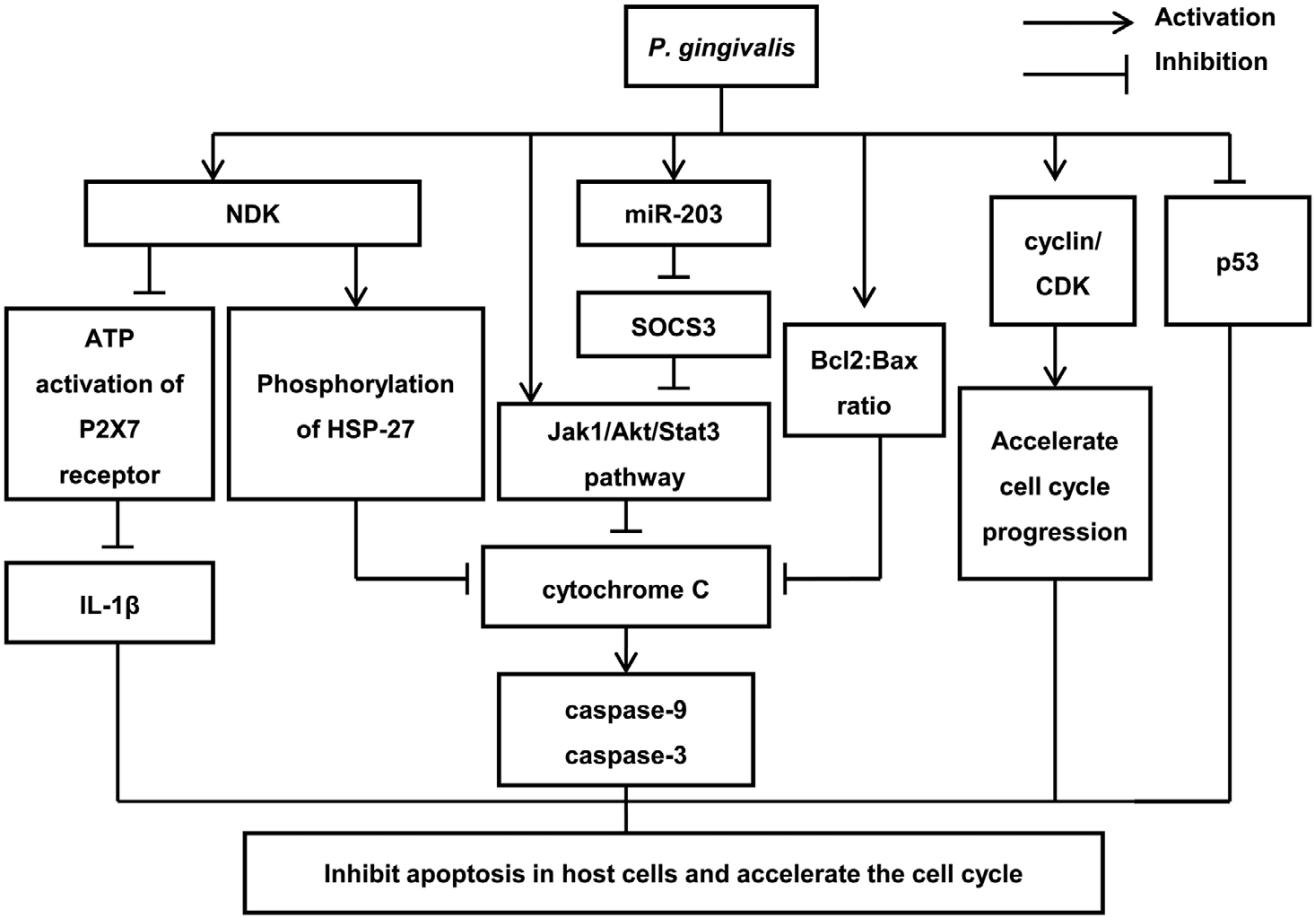
The mechanism by which *Porphyromonas gingivalis* inhibits apoptosis in host cells and accelerates the cell cycle.

*P. gingivalis* inhibits apoptosis through various mechanisms in primary epithelial cells (Fig. 3). (1) *P. gingivalis* secretes nucleoside diphosphate kinase (NDK) after entering human cells, which has a similar function to adenosine triphosphate (ATP). NDK from *P. gingivalis* antagonises ATP activation of P2X7 receptors, and thus reduces IL-1β production from epithelial cells, which inhibits ATP-dependent apoptosis and increases the incidence of cancer [52, 53]. Additionally, NDK phosphorylation of heat shock protein-27 (HSP-27) curtails cytochrome C release and caspase-9 activation, thus stalling apoptosis [54]. (2) At the mitochondrial membrane, *P. gingivalis* inhibits apoptosis by activating the Janus kinase 1(Jak1)/threonine kinase (Akt)/signal transducer and activator of transcription 3 (Stat3) signalling pathways, reducing the content of the proapoptotic protein Bad, increasing the ratio of the antiapoptotic factor Bcl2 protein to the proapoptotic factor Bax, decreasing the release of the apoptosis effector cytochrome oxidase C, and inhibiting downstream caspases-3 activation [55, 56]. Our previous study verified that *P. gingivalis* infection reduces the sensitivity of ESCC cells to chemotherapy drugs by activating the Stat3 signalling pathway in vivo and in vitro [57]. Compared with the control group, *P. gingivalis* infection significantly enhanced Stat3 expression and inhibited caspase-3 expression in paclitaxel-treated KYSE-30 cells. These results suggest that differential regulation of Stat3 and caspase-3 is the key pathway involved in the resistance of oesophageal cancer cells to apoptosis induced by *P. gingivalis* infection. (3) *P. gingivalis* upregulates the expression level of miRNA-203, which results in the decreasing levels of suppressor of cytokine signalling 3 (SOCS3) and subsequently suppresses apoptosis of primary gingival epithelial cells [58]. Since SOCS3 can bind to phosphorylated JAK receptors, it consequently inhibits JAK/STAT3 signalling [59]. In addition, *P. gingivalis* regulates the activity of cyclin/cyclin-dependent kinase (CDK), accelerating the progression of primary gingival epithelial cells through the cell cycle. Besides, *P. gingivalis* also reduces the level of the tumour suppressor p53, leading to either progression of cell cycle, or to inhibition of apoptosis [60] (Fig. 3).

## Summary and outlook

There is a high incidence of oesophageal cancer in China, and the morbidity and mortality of this disease are much greater in China than the world’s average. Overall, oesophageal cancer is a major public health problem that urgently needs to be addressed. Although previous studies have shown that *P. gingivalis* may be closely related to the occurrence and development of oesophageal cancer, tumour occurrence is the result of the synergistic effects of multiple factors. Whether *P. gingivalis* can be exclusively pathogenic or has related synergistic pathogenic factors and how these factors function need to be further explored. Most studies to date have only focused on the correlation between *P. gingivalis* and oesophageal cancer, and there are still many gaps in our knowledge of the aetiology, pathology, and immunology. Therefore, further research is needed to prevent and treat oesophageal cancer by monitoring oral bacteria and to identify therapeutic targets through relevant mechanisms.

### Early monitoring of oesophageal cancer by studying changes in the oral flora

Through prospective cohort studies, the changes in the specific oral microbes or biomarkers related to oesophageal cancer will be further clarified, which is expected to transform basic research on the oral flora into a new technology to predict the risk of oesophageal cancer and provide personalised early risk warning services for patients. Moreover, the identification of specific oral bacteria associated with oesophageal cancer can both promote our understanding of the disease aetiology and provide biomarkers for oesophageal cancer occurrence and development. Of note, oral bacterial isolation and detection are relatively inexpensive and noninvasive, and could provide a new indicator for diagnosing tumours. The convenience of *P. gingivalis* detection can help to identify high-risk groups and enable the monitoring and prevention of related diseases.

### Assisting in the prevention and clinical treatment of oesophageal cancer

After the mechanism by which *P. gingivalis* affects the occurrence and development of oesophageal cancer is clarified, targeted therapy can be implemented by eliminating *P. gingivalis* or targeting specific cell adhesion sites, inhibitory immune receptors, cell pathways, and cytokines to achieve oesophageal cancer treatment. In addition, new therapies have been developed to treat or prevent malignant tumours by regulating the intestinal microbiota through microbial therapies to better block tumour progression. For example, the use of antibiotics or the transplantation of probiotics to maintain the homeostasis of relevant tissue sites is important for preventing cancer in high-risk populations. The measurement of *P. gingivalis* antibody levels to assess the risk of disease, lesion degree, and prognosis is conducive to the formulation of clinical strategies to achieve a better treatment effect in oesophageal cancer. Furthermore, since the presence of *P. gingivalis* may impact the efficacy of chemotherapy due to the interaction between the microbiota and immune system, it can be used as a predictor of adverse chemotherapy reactions and efficacy [57]. However, given the relationship between the complexity of the microbial groups in the human body and tumour cells, interdisciplinary research incorporating oncology, microbiology, immunology, and pathology is needed if the characteristics of *P. gingivalis* are to be applied in clinical microbial therapy. Based on this analysis, oral bacterial groups can be used as targets to formulate more valuable strategies for the prevention and treatment of malignant tumours.

In conclusion, *P. gingivalis* is related to the occurrence and development of oesophageal cancer, but a large sample, multicentre prospective cohort study is still needed to further explore the pathogenesis of *P. gingivalis* to provide new ideas for the prevention and treatment of oesophageal cancer and improve the health and quality of life of patients.
